# Differential Effects of β-Blockers, Angiotensin II Receptor Blockers, and a Novel AT2R Agonist NP-6A4 on Stress Response of Nutrient-Starved Cardiovascular Cells

**DOI:** 10.1371/journal.pone.0144824

**Published:** 2015-12-21

**Authors:** Abuzar Mahmood, Lakshmi Pulakat

**Affiliations:** 1 Department of Medicine, University of Missouri, Columbia, MO, United States of America; 2 Department of Nutrition and Exercise Physiology, University of Missouri, Columbia, MO, United States of America; 3 Harry S. Truman Memorial Veterans’ Affairs Hospital, Columbia, MO, United States of America; University of Sassari, ITALY

## Abstract

In order to determine differences in cardiovascular cell response during nutrient stress to different cardiovascular protective drugs, we investigated cell responses of serum starved mouse cardiomyocyte HL-1 cells and primary cultures of human coronary artery vascular smooth muscles (hCAVSMCs) to treatment with β-blockers (atenolol, metoprolol, carvedilol, nebivolol, 3μM each), AT1R blocker losartan (1μM) and AT2R agonists (CGP42112A and novel agonist NP-6A4, 300nM each). Treatment with nebivolol, carvedilol, metoprolol and atenolol suppressed Cell Index (CI) of serum-starved HL-1 cells (≤17%, ≤8%, ≤15% and ≤15% respectively) as measured by the Xcelligence Real-Time Cell Analyzer (RTCA). Conversely, CI was increased by Ang II (≥9.6%), CGP42112A (≥14%), and NP-6A4 (≥25%) respectively and this effect was blocked by AT2R antagonist PD123319, but not by AT1R antagonist losartan. Thus, the CI signature for each drug could be unique. MTS cell proliferation assay showed that NP-6A4, but not other drugs, increased viability (≥20%) of HL-1 and hCAVSMCs. Wheat Germ Agglutinin (WGA) staining showed that nebivolol was most effective in reducing cell sizes of HL-1 and hCAVSMCs. Myeloid Cell Leukemia 1 (MCL-1) is a protein critical for cardiovascular cell survival and implicated in cell adhesion. β-blockers significantly suppressed and NP-6A4 increased MCL-1 expression in HL-1 and hCAVSMCs as determined by immunofluorescence. Thus, reduction in cell size and/or MCL-1 expression might underlie β-blocker-induced reduction in CI of HL-1. Conversely, increase in cell viability and MCL-1 expression by NP-6A4 through AT2R could have resulted in NP-6A4 mediated increase in CI of HL-1. These data show for the first time that activation of the AT2R-MCL-1 axis by NP-6A4 in nutrient-stressed mouse and human cardiovascular cells (mouse HL-1 cells and primary cultures of hCAVSMCs) might underlie improved survival of cells treated by NP-6A4 compared to other drugs tested in this study.

## Introduction

Cardiovascular diseases, particularly ischemic heart disease, are the number one cause of death world-wide despite commendable advances in acute care and pharmacotherapy [1–4]. Cardiomyocyte death via necrosis, apoptosis and impaired autophagy are hallmarks of cardiac pathology associated with heart failure, myocardial infarction and ischemia/reperfusion injury [3–6]. Anti-hypertensive drugs such as β-adrenergic receptor blockers (β-blockers) and inhibitors of angiotensin II type 1 receptor (AT1R) are reported to exert cardioprotective effects by reducing cardiomyocyte death [7–11].

β-adrenergic receptor blockers (β-blockers) are the standard of care for myocardial infarction (MI) and ischemic heart disease. However, recent clinical trials have questioned the morbidity and mortality benefits of these drugs in the management of patients with cardiac disease [12–14]. Traditional contraindications for β-blockers include peripheral vascular diseases, diabetes mellitus, chronic obstructive pulmonary disease (COPD) and asthma [12–14]. The 2^nd^ generation β-blockers atenolol (Aten) and metoprolol (Met) are more likely to worsen glucose tolerance and increase the risk of developing diabetes [15, 16]. The 3^rd^ generation β-blockers carvedilol (Car) and nebivolol (Neb) are considered to be safer and more effective drugs since Car blocks the α-adrenergic receptor and improves vasodilation, and Neb activates the cardioprotective β-3 adrenergic receptor that results in activation of the AMP kinase (AMPK)-endothelial Nitric Oxide Synthase (eNOS) pathway [10,17–20]. Neb might function as a biased agonist and could reduce weight gain in rodents and humans [18–20]. We have shown recently that Neb–induced resistance to weight gain in leptin resistant rats involves the cardiac miR-208-MED13 axis [21]. However, further studies are needed to fully understand the protective effects of Neb compared to other β-blockers on cardiovascular cells subjected to nutrient stress.

Angiotensin II (Ang II) acting through the AT1R is an important contributor to vasoconstriction and promotes cardiac hypertrophy, fibrosis and heart disease [22, 23]. Moreover, AT1R activation induces adult cardiomyocyte cell death [24, 25]. AT1R blockers (ARBs) are another group of widely used drugs to treat patients with hypertension, atherosclerosis, coronary heart disease, restenosis, and heart failure. However, clinical trials have raised concerns regarding the potential of ARBs to increase risk of MI [26]. Unlike AT1R, activation of Ang II type 2 receptor (AT2R) causes vasodilation and improves cardiac repair after MI [27, 28]. We have shown that AT2R activation can inhibit AT1R-mediated inositol 1,4,5-triphosphate generation and that the 3^rd^ intracellular loop of AT2R is required for this effect [29]. Though AT2R activation causes neonatal cardiomyocyte apoptosis, this effect is not seen in adult cardiomyocytes [30, 31]. However, signaling mechanisms of the AT2R are less defined compared to that of the AT1R and drugs that can act as specific AT2R agonists are still emerging.

Serum starvation that results in nutrient deficiency stress is an important factor associated with ischemic heart disease and contributes to significant loss of cardiovascular cells via cell death [32, 33]. To gain a better understanding of the potential of different cardioprotective drugs to improve cardiovascular cell survival during nutrient deficiency stress, we compared the effects of different cardioprotective drugs on cell survival of mouse cardiomyocyte HL-1 cells and primary cultures of human coronary artery vascular smooth muscle cells (hCAVSMCs) subjected to serum starvation. For studies on HL-1 cells, we used the xCELLigence RTCA (Real-Time Cell Analyzer), a system that provides an effective method to assess survival and adhesion properties of cells by obtaining real-time kinetic data that captures an accurate characterization of short-lived changes in cell size, number and adhesion [34,35]. This system measures real-time electrical impedance variations in microelectrodes at the base of 16-well microtiter “E-plates” and reports it in terms of cell index (CI). We hypothesized that although all drugs would protect adult cardiovascular cells from significant cell death, the CI pattern or the “CI signature” generated by these drugs could be different and would help to explain which of these drugs most effectively renders protection during nutrient starvation stress. Therefore, we investigated the differences in the CI signatures of serum starved mouse atrial cardiomyocyte HL-1 cells treated with different drugs (Table 1). The β-blockers used in this study were Aten, Met, Car and Neb. In addition, we tested the effect of β-3 AR specific blocker SR59230A. To determine the effects of activating the Ang II receptors, we investigated how Ang II, the AT2R partial agonist CGP42112A (CGP), and a patent-pending novel peptide agonist of the AT2R named NP-6A4 (developed by Novopyxis Inc.) modulate CI. To further confirm the involvement of specific AngII receptors (AT1R or AT2R) we also used AT1R antagonist losartan (Lo) and AT2R antagonist PD123319 (PD) in conjunction with the agonists.

**Table 1:** List of drugs used in this study and their targets.

Drug Used	Known Targets	Mode of Action
Nebivolol	β1 AR; β3 AR	Antagonist; Agonist
Carvedilol	β1 and β2 AR	Antagonist
Metoprolol	β1 and β2 AR	Antagonist
Atenolol	β1 and β2 AR	Antagonist
Angiotensin II	AT1R and AT2R	Agonist
CGP42112A	AT2R	Partial Agonist
NP-6A4	AT2R	Agonist
Losartan	AT1R	Antagonist
PD123319	AT2R	Antagonist
SR59230A	β3 AR	Antagonist
U73122	Phospholipase C	Inhibitor

To further determine the contribution of cell viability and cell size to CI, we investigated changes in cell proliferation and viability of serum starved HL-1 in response to above drug treatments by MTS [3-(4,5-dimethylthiazol-2-yl)-5-(3-carboxymethoxyphenyl)-2-(4- sulfophenyl)-2H-tetrazolium] Cell Proliferation Assay. Changes in cell size of serum starved HL-1 in response to drug treatments were assessed by staining with Wheat germ agglutinin (WGA) conjugated with Alexa Fluor ^®^ 647. To verify whether drug responses of mouse HL-1 cells, an immortalized cell line, could be translational in human cells, we examined changes in cell viability of primary cultures of hCAVSMCs in response to the above drug treatments by MTS Cell Proliferation Assay. We also determined changes in cell sizes of nutrient-stressed hCAVSMCs in response to drug treatments by staining with WGA conjugated with Alexa Fluor ^®^ 647.

Myeloid Cell Leukemia 1 (MCL-1) is a protein that is essential for cell survival and viability of cardiomyocytes and VSMCs. Loss of MCL-1 in cardiac tissue results in cardiomyocyte disorganization, rapid heart failure and mitochondrial dysfunction [36–38]. MCL-1 is also implicated in VSMC survival [39]. Moreover, MCL-1 is required for cell adhesion [40]. We posited that increase in MCL-1 would improve viability of HL-1 and hCAVSMCs. Therefore, we investigated whether the drugs that were most effective in increasing the CI of HL-1 cells could also increase MCL-1 expression in HL-1 and hCAVSMCs.

## Materials and Methods

### Cell Culture and Reagents

Mouse atrial cardiomyocyte HL-1 cells were a gift from Dr. William Claycomb at Louisiana State University Medical Center. HL-1 cells were cultured at 37°C and 5% CO_2_ on surfaces pre-treated with 12.5 μg/ml bovine fibronectin in 0.02% gelatin solution and grown in Claycomb medium supplemented with Fetal Bovine Serum (10%) (both from Sigma-Aldrich, St. Louis, MO), Penicillin/Streptomycin (100U/ml: 100mg/ml), Norepinephrine (100U/ml) and L-Glutamine (2mM) (GIBCO-Invitrogen) as described previously [38, 41]. hCAVSMCs were purchased from GIBCO-Invitrogen Cell culture (Carlsbad, CA) and were cultured according to manufacturer’s instructions at 37°C and 5% CO_2_ in Medium 231 supplemented with Smooth Muscle Growth Supplement (SMGS, Life Technologies, Cat. No. S-007-25). β-blockers Aten, Met and Car and Ang II were purchased from Sigma-Aldrich, and Neb was a gift from Forest Laboratories Inc. (New York). Losartan (AT1R inhibitor), PD123319 (AT2R inhibitor), CGP42112a (partial AT2R agonist), SR59230A (β-3 AR specific blocker), U73122 (Phospholipase C inhibitor) and isoproterenol (β-AR activator) were purchased from Tocris Bioscience (Bristol, UK); NP-6A4 was a gift from Novopyxis Inc. (Boston).

### The xCELLigence RTCA and characterization of CI signatures of HL-1 cells

E-plates treated with fibronectin [38, 41] were used to seed HL-1 cells and to determine the changes in cell index readings in response to different drug treatments by RTCA DP Instrument (from ACEA Biosciences Inc.). A series of pilot studies were performed to determine the number of cells to be seeded and the time required for cell attachment before adding the drugs to obtain a consistent starting CI for all wells (data not shown). Based on the results of these pilot studies, we seeded 1.6 x 10^4^ cells in each well of a 16-well E-plate in complete Claycomb Medium for all studies reported here and initiated monitoring of CI every 15 minutes. Cell attachment was assessed by stabilization of CI and confirmed by visualization under a light microscope (Nikon Eclipse TS1000). Once cells attached, wells were washed twice with serum-free medium and 95μL of serum-free medium was added to each well. CI was monitored at 5 min intervals to ensure that CI was stabilized before adding the drugs. Then, 5μL of each drug was added to achieve appropriate final concentrations. CI was monitored at every 5 min for the next 24 to 48 hours. Final concentrations for Aten, Met, Car, Neb and SR59230A (β-3 AR specific blocker) were 3μM since 3 μM was the lowest concentration used to show vasorelaxant activity of Neb and concentration of other β-blockers were kept 3μM for comparison [42]. Final concentrations for PD123319 and losartan were 1μM since we have previously shown effects of losartan and PD123319 at this concentration successfully [43]. Ang II, CGP42112A and NP-6A4 were used at a final concentration of 300nM since Ang II is often used at a final concentration of 300nM for vasoconstriction studies [44] and also to ensure that the concentration used is lower than that of the antagonists. Data reported is from a minimum of three independent E-plate experiments (different passages of HL-1 cells) and in each experiment, a given drug treatment was performed at least in triplicates and in wells at different locations to ensure that the data was not affected by positional effects.

### Cell viability assay

MTS [3-(4,5-dimethylthiazol-2-yl)-5-(3-carboxymethoxyphenyl)-2-(4- sulfophenyl)-2H-tetrazolium] Cell Proliferation Assay kit (Biovision Inc) was used according to the manufacturer’s instructions to determine the effects of different drugs on the cell viability of HL-1 cells and hCAVSMCs. HL-1 or hCAVSMC cells were seeded (5 x 10^3^cells/well) in 96 well plates in complete Claycomb medium [38] or Medium 231 supplemented with SMGS, respectively and all incubations were performed at 37°C in the presence of 5% CO_2_. After confirming that the cells were attached (as determined by light microscopy analysis), culture medium was removed and 200μL of serum-free Claycomb medium was added to HL-1 cells and 200μL of medium 231 without SMGS was added to CAVSMCs. Cells were then subjected to drug treatments by following a time course identical to those used for the xCELLigence RTCA. At the end of treatment, 20μL of MTS was mixed with culture medium and absorbance was measured at 30 min intervals at 490nm using the Synergy H4 Hybrid plate reader (BioTek, Vinooski, VT). Data is presented as the percentage of absorbance in drug treated cells compared to untreated cells.

### Immunofluorescence

Immunofluorescence was used to determine the changes in the expression of MCL-1 in HL-1 cells and hCAVSMCs in response to different treatments. HL-1 cells and hCAVSMCs were grown on cover slips. All drug treatments were performed at least in triplicates and treatments were performed for six hours to match the time course in which we saw significant differences in the CI in response to drug treatments. Concentrations of drugs used for treatments were exactly the same as those used for xCELLigence RTCA analysis. After treatments with drugs, coverslips were washed with HEPES (Sigma), fixed with 4% paraformaldehyde for 15 min at room temperature, permeabilized with 0.5% Triton X-100, washed with HEPES buffer, and blocked with 1% bovine serum albumin (BSA) (Jackson ImmunoResearch), along with 10% goat serum (Sigma) in HEPES-T (1mL Tween-20/L). Coverslips were then incubated with anti-MCL-1 antibody (Abcam) (1:100 dilution, ~15 μg/ml) overnight at 4°C, washed with HEPES and then incubated with Alexa Fluor 488 goat anti-rabbit antibody (Invitrogen, Inc.) (1:200 dilution, 10 μg/ml) for 1 hour at room temperature. The coverslips were then washed with HEPES and mounted with Fluoroshield with DAPI (4',6-diamidino-2-phenylindole) (Sigma-Aldrich).

Wheat germ agglutinin (WGA) conjugated with Alexa Fluor ^®^ 647 (Invitrogen, Inc.) was used to label HL-1 and hCAVSMC cell membranes and was used according to manufacturer’s instructions. HL-1 cells were grown on coverslips in the presence of Claycomb medium containing 0.5% FBS (HL-1) for 48 hours and then the drug treatments were performed for 6 and 24 hours. For hCAVSMCs, drug treatments were performed for 6 hours and the cells were maintained in Medium 231 without SMGS. At the end of treatments, cells were fixed and stained. Visualization was performed using a Leica DMI 4000B inverted confocal microscope using Leica Application Suite software. Imaging was done at 40x and 63x magnification using oil immersion. Fluorescence intensity and cell size measurement was done using ImageJ software (NIH, Bethesda, MD).

### Statistics

Statistical analysis was performed using the SPSS 20 software package. Results are expressed as mean ± SEM (standard error of mean). Differences between groups were tested by using one-way ANOVA followed up with the Least Significant Difference (LSD) post-test or t-test, as appropriate, and two-tailed p-values are reported. A *p*-value of ≤ 0.05 was considered statistically significant.

## Results

### CI signature of nebivolol and carvedilol are different from that of atenolol and metoprolol

**Fig 1:**
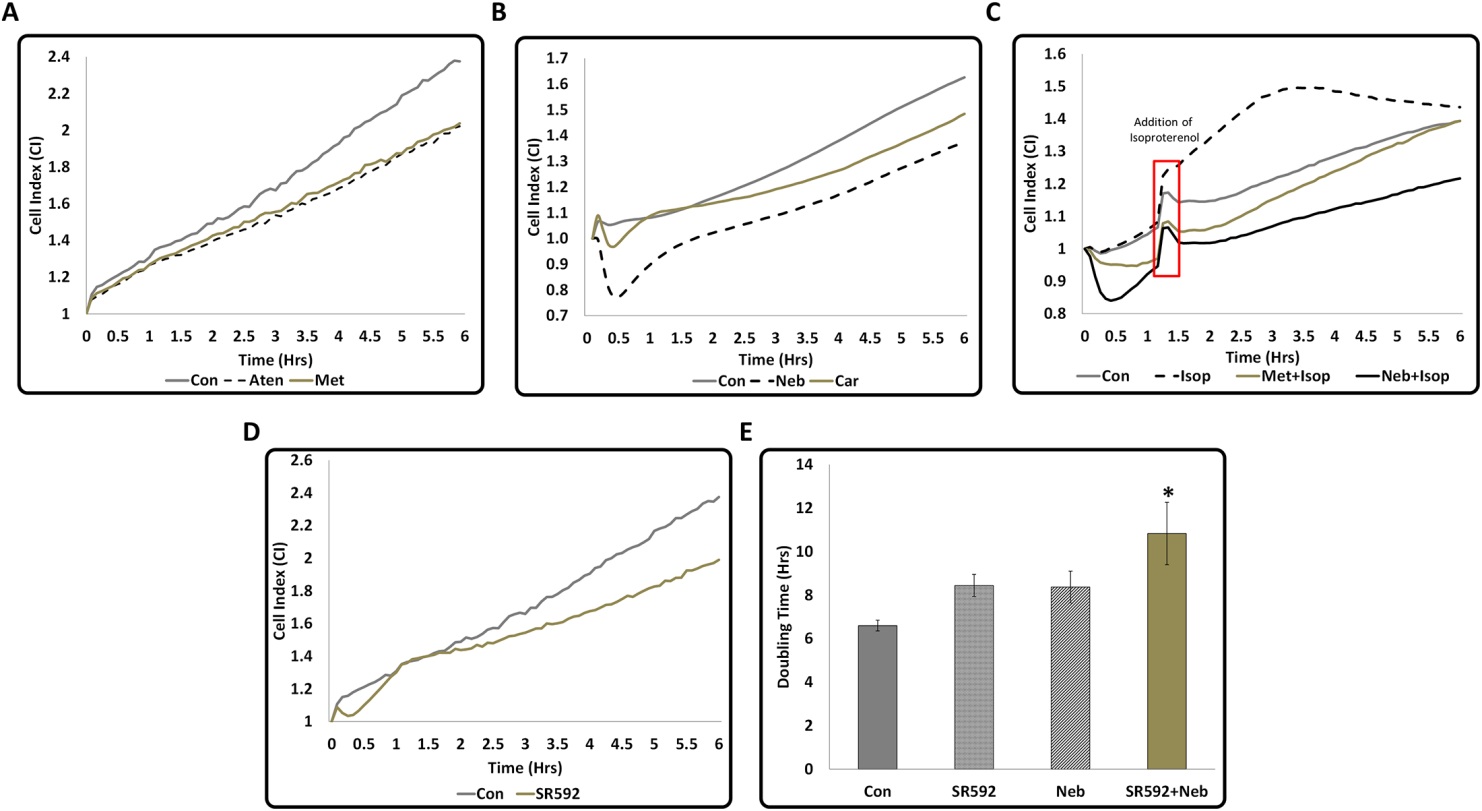
Differences in the CI signatures of 2^nd^ and 3^rd^ generation β-Blockers, Isoproterenol, and the β3-AR inhibitor SR59230A in serum-starved HL-1 cardiomyocytes. Cell Index (CI) data generated by Xcelligence RTCA when serum starved HL-1 cells were treated with different drugs for six hours are shown in graphs (A-D). All drugs except isoproterenol reduced the rate of increase in CI with time. Since all drugs were dissolved in Dimethyl sulfoxide (DMSO), control cells in this experiment received 0.05% DMSO (similar to the amount of DMSO in the drug preparation). **(A)** Changes in the CI of 2^nd^ generation β-Blockers Aten (300μM) and Met (300μM) were similar. **(B)** Changes in the CI of 3^rd^ generation β-Blockers Neb and Car were different from that that of the 2^nd^ generation β-Blockers and also different from each other. A quick suppression (a dip) of CI was observed within 30 minutes of addition of these drugs. This was not observed with the 2^nd^ generation β-Blocker treatments. Suppression of CI was more pronounced with Neb than Car indicating that each drug had unique CI signature. **(C)** Changes in CI in response to Isoproterenol (Isop) (added after one hour pre-treatment with Neb or Met). The red box indicates the time taken for addition of isoproterenol and the change in the pattern of graph due to the brief temperature change since the door was opened to add the drug. Isoproterenol increased CI initially compared to Con, however, after 2 hours, there was a reduction in CI. **(D)** Changes in CI in response to β3-AR inhibitor SR59230A exhibiting an initial sudden dip. Values are shown as means, n≥4 and p<0.05 for all treatments compared to control for (A-D). **(E)** Combination of Neb and SR59230A increases CI doubling time compared to treatment by either drug alone, suggesting further suppression of cell growth. Values are means ± SEM for each point in the graphs, n = 4, *p<0.05 compared to control. All statistical significance determined by one-way ANOVA followed by the LSD post-hoc test.

All β-blockers are known to inhibit cardiomyocyte apoptosis [7–10]. However, changes in CI over time in response to treatment with different β-blockers were not similar. HL-1 cells treated with Aten and Met exhibited a similar pattern of changes in CI and their CIs were lower by ~15% than the CI of untreated cells (Fig 1A). Changes in the CI of HL-1 cells treated with Car exhibited an initial suppression followed by a recovery, however, overall, CI still remained lower by ~8% than that of untreated cells (Fig 1B). Neb-treated cells showed a much steeper reduction in CI (~20%) within the first 30 min of treatment followed by a recovery, but Neb treatment resulted in the lowest CI (~17%;Fig 1B). These data suggested that CI signatures generated by Neb and Car are unique and different from the CI signatures of Met or Aten. Conversely, isoproterenol (Isop), a standard selective β-adrenoceptor agonist, increased the CI immediately after addition when used at a concentration of 50nM and this increase (~22%) was maximum after 3 hours (Fig 1C). To determine the effect of inhibition of β3-AR, we treated HL-1 cells with SR59230A which also showed a reduction in CI (~15.5% at 6 hour time point) (Fig 1D). Moreover, exposure of HL-1 cells to a combination of SR59230A and Neb appears to increase the doubling time of the CI even further than either drug individually, indicating that the cell survival or growth was further impaired (Fig 1E). Collectively these data suggest that blocking of the β-ARs expressed by HL-1 cells have an inhibitory effect on their CI.

### Inhibition of phospholipase C reduces the CI of HL-1 cells

**Fig 2:**
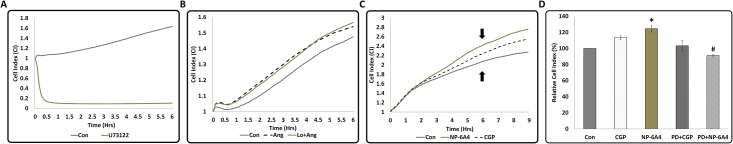
Effect of phospholipase C inhibition and Angiotensin II Type 2 receptor (AT2R) activation on CI of serum-starved HL-1 cardiomyocytes. **(A)** Treatment by PLC inhibitor U73122 resulted in a drastic drop in CI immediately after adding the drug. **(B)** Treatment by Angiotensin II (Ang II) increased CI compared to Con and pre-treatment with losartan (Lo), an AT1R antagonist did not reduce Ang II-induced increase in CI. Therefore, AT1R activation by Ang II is not responsible for Ang II induced increase in CI. **(C)** Treatment by AT2R specific agonists increased CI with magnitude of increase of CGP42112A<NP-6A4. CGP42112A is referred as CGP in the graphs. Since difference between CI of the control (treated with saline) and drugs was maximum at about 6 hours, the 6 hour time point was selected for final comparison shown in Fig D. Values are means ± SEM for each point in the graphs, n = 4, *p<0.05 compared to control for graphs A-C. **(D)** CI at 6-hour time-point showing pre-treatment by AT2R specific inhibitor PD123319 abolishes the effect of CGP42112A and NP-6A4; therefore, the CI increase by these agonists is mediated by AT2R. PD123319 is referred as PD in the graphs. Values are means ± SEM, n≥4 and *p<0.05.

Previous studies have shown that phospholipase Cγ is essential for cardiomyocyte survival under oxidative stress, and inhibition of phospholipase Cγ with U73122 increases apoptosis [43]. Since nutrient starvation can induce oxidative stress, we tested whether inhibition of phospholipase Cγ by U73122 in the serum-starved HL-1 cells results in cytotoxicity. Treatment of serum-starved HL-1 cells with U73122 suppressed their CI drastically (~94%) (Fig 2A). This observation suggests that inhibition of phospholipase Cγ under serum starvation promotes cytotoxicity and this can be detected by reduction in CI.

### Ang II increased CI of HL-1 cells and AT1R antagonist did not suppress this effect

Ang II acting through AT1R promotes cell growth [22, 23, 29]. To determine the effects of Ang II on the CI unit signature of HL-1 cells, we investigated the changes in CI of HL-1 cells treated with Ang II (300nM) in the presence and absence of AT1R inhibitor losartan (1μM). The CI of HL-1 cells treated with Ang II was higher than that of untreated HL-1 cells (≥9.6%) and pretreatment with losartan did not change this effect (Fig 2B). This data indicates that Ang II-induced increase in CI is not mediated through AT1R.

### Treatment with NP-6A4, a novel peptide agonist of AT2R, resulted in the highest increase in CI of HL-1 cells

Since Ang II also activates the AT2R, to determine the effects of AT2R activation on the CI signature of HL-1 cells, we determined changes in CI of HL-1 cells treated with a partial AT2R agonist, CGP42112A [45], and a novel AT2R agonist, NP-6A4. NP-6A4 is a small peptide, anti-inflammatory drug that binds AT2R according to computational modeling and simulation (from Novopyxis, Inc. patent pending). Treatments with CGP4211A and NP-6A4 (300nM of each drug) showed a significant increase in the CI (CGP42112A:≥14%, NP-6A4:≥25%) of HL-1 cells (Fig 2C). NP-6A4 treatment resulted in the highest increase in CI of HL-1 cells by the 6 hour time point (Fig 2C). To confirm that the NP-6A4-induced increase in CI of HL-1 cells is due to AT2R activation, we tested whether this effect is inhibited by the AT2R specific antagonist PD123319. Pre-treatment with 1μM of PD123319 inhibited increase in CI induced by CGP42112A and NP-6A4 (Fig 2D). These data suggest that AT2R activation results in an increase in CI of HL-1 cells and that NP-6A4 increases the CI of HL-1 cells by activating AT2R.

### Changes in Cell size of HL-1 cells in response to drug treatments

**Fig 3:**
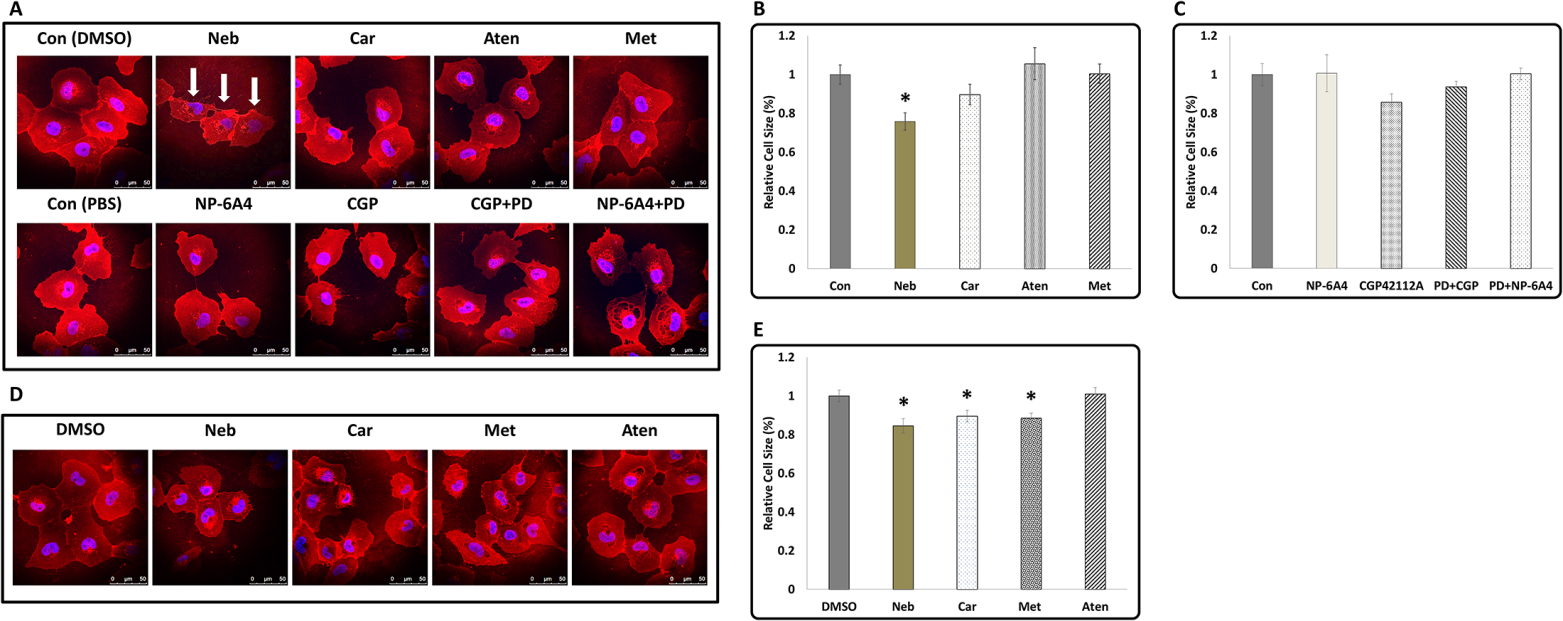
The effect of β-Blockers and AT2R agonists on cell size in HL-1 cardiomyocytes. **(A)** Representative images of serum starved HL-1 cells subjected to treatments with beta blockers (top panel) or AT2R agonists (bottom panel) for 6 hours and visualized by WGA staining and nuclear staining with DAPI (scale bars = 50μm). Reduction in cell size after treatment with Neb (marked by white arrows) was visible after 6-hour treatment. (**B and C**) Quantification of the area of stained cells in Fig A. **(D)** Representative images of serum starved HL-1 cells subjected to treatments with only beta blockers for 24 hours and visualized by WGA staining and nuclear staining with DAPI (scale bars = 50μm). Met and Car also reduced cell size when treatment was continued for 24 hours. **(E)** Quantification of the area of stained cells in Fig D. Values are means ± SEM, n≈20 for each treatment, *p<0.05. All statistical significance determined by One-way ANOVA followed by the LSD post-hoc test.

To determine whether there was any difference in the cell sizes of HL-1 cells in response to drug treatments, we stained untreated and drug-treated cells with wheat germ agglutinin (WGA) and measured their size using ImageJ software (NIH, Bethesda, MD). Cells were initially made quiescent by growing them on coverslips in the presence of Claycomb medium containing 0.5% FBS for 48 hours and then the drug treatments were performed for a period of 6 and 24 hours [46]. WGA staining at the end of six-hour treatment showed that Neb significantly reduced cell size (~25%) while other drug treatments did not have a significant effect (Fig 3A–3C). However, when the drug treatments were extended for 24 hours, treatments with Met and Car also resulted in a significant reduction in cell size (Fig 3D and 3E). Treatment with Aten did not change cell size (Fig 3B and 3E). This observation suggested that changes in cell size could be one of the mechanisms for reduction in CI in response to treatments with Neb, Met and Car.

### NP-6A4 improved cell viability of serum starved HL-1 cells

**Fig 4:**
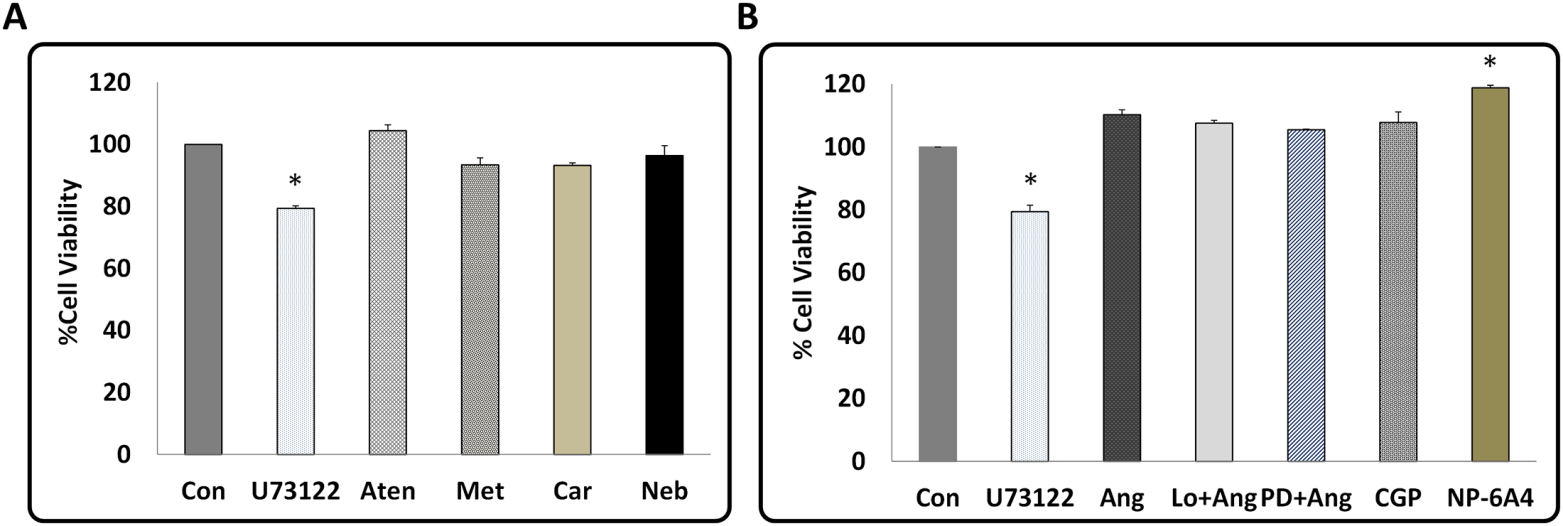
Effect of β-Blockers, Angiotensin II, AT2R agonists, and AT1R- and AT2R-antagonists on cell viability in HL-1 cardiomyocytes. Changes in cell viability of serum starved HL-1 cardiomyocytes in response to treatment by U73122 and β-Blockers (A), and Ang II, Ang II+ losartan, Ang II+ PD123319, and AT2R agonists (B) as determined by the MTS proliferation assay. **(A)** As expected, phospholipase C inhibition by U73122 showed a strong suppression of cell viability. Treatment with β-Blockers did not result in any significant difference in cell viability compared to DMSO treated control. **(B)** Treatment NP-6A4 showed the greatest increase in cell viability. Data for A and B are presented as means ± SEM, n≥3 for all treatments, *p<0.05 compared to control as determined by One-way ANOVA followed by the LSD post-hoc test.

To determine the correlation between CI and cell viability of HL-1 cells, the MTS cell proliferation assay was used. When compared to untreated, serum starved HL-1 cells, treatment with U73122 reduced cell viability (~20%) and this effect was statistically significant (Fig 4A). U73122 also decreased the CI of HL-1 cells drastically (Fig 2A). Thus, reduction in cell viability could, in part, account for the drastic reduction in CI of HL-1 cells by U73122. None of the β-blockers significantly changed the cell viability of serum starved HL-1 cardiomyocytes (Fig 4A). Thus, the suppression of CI induced by the β-blockers is not due to a reduction in cell viability, but rather other mechanisms. Interestingly, AT2R activation by NP-6A4 increased cell viability of serum starved HL-1 cells significantly when compared to untreated cells (Fig 4B). Therefore, increase in cell viability could have contributed to increase in CI by NP-6A4.

### NP-6A4 increased the expression of MCL-1 in HL-1 cells

**Fig 5:**
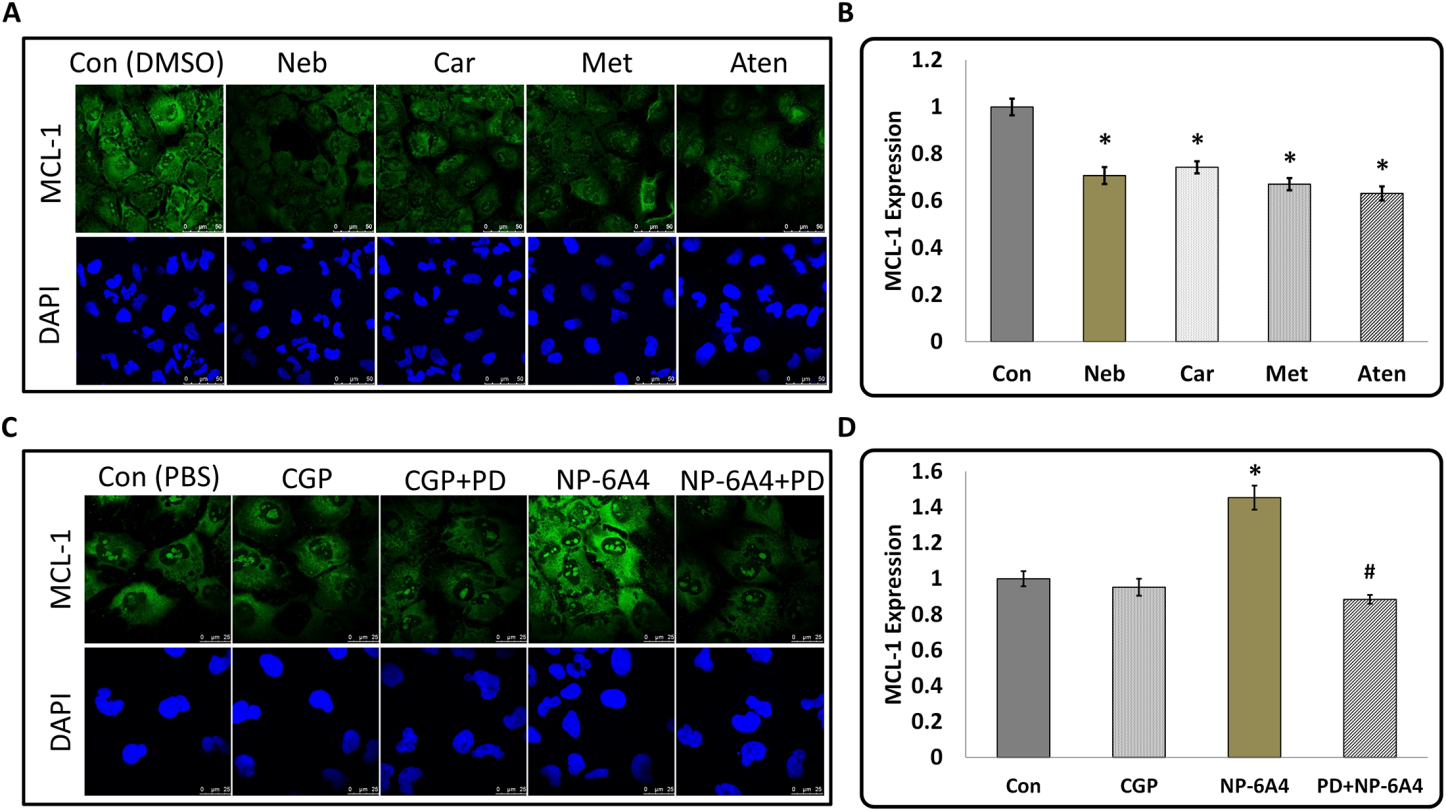
Effects of β-Blockers, and AT2R agonists and antagonists on the expression of anti-apoptotic protein Myeloid Cell Leukemia 1 (MCL-1) in HL-1 cardiomyocytes. **(A and C)** Representative images of immunofluorescence staining with anti-MCL-1 antibody and nuclear stain DAPI of HL-1 cardiomyocytes in response to treatment by β-Blockers, and AT2R agonists in the presence and absence of AT2R antagonist PD123319 (scale bars = 50 μm for A and 25μm for C). **(B and D)** Graphs show quantification of MCL-1 expression in response to treatment with drugs as marked. MCL-1 expression was suppressed in response to treatment by all β-Blockers. Novel agonist NP-6A4 increased MCL-1 expression and this effect was abolished by pretreatment with AT2R specific inhibitor PD123319. Since MCL-1 promotes cell survival under stress [36, 37], these findings implicate the involvement of MCL-1 in CI changes in response to treatment by β-Blockers and AT2R agonists. n≈50 for all treatments, *p<0.05 compared to respective control and #p<0.05 compared to NP-6A4 as determined by Student’s 2-tailed T-test.

MCL-1 is a critical molecule for cardiomyocyte survival, since ablation of cardiac specific MCL-1 causes fatal heart failure and significant mitochondrial damage [36, 37]. We have recently reported that suppression of cardiac MCL-1 expression correlates with cardiomyocyte disarray in diabetic rats [38]. It is also known that MCL-1 may be important for cell adhesion [40]. Since CI is a measure of cell number, cell size and adhesion, and MCL-1 is involved in maintaining cell viability and adhesion, we posited that expression of MCL-1 would correlate with the CI. To determine whether MCL-1 expression in HL-1 cells correlated with the changes in their CIs in response to treatments with β-blockers and NP-6A4, we investigated the changes in MCL-1 expression in HL-1 cells subjected to treatment with these drugs. Drug treatments were performed for 6 hours since at this time point drug treatment yielded significant difference between the CI of drug-treated and vehicle-treated cells. As presented in Fig 5A and 5B, immunofluorescence analysis of MCL-1 expression and quantification using ImageJ (~50 cells per treatment) showed that treatments with all β-blockers suppressed MCL-1 expression significantly (Neb≤29%, Car≤25%, Met≤32% and Aten≤36%). Conversely, AT2R agonist NP-6A4 increased MCL-1 expression by ≥45% (Fig 5C). This effect was inhibited by the AT2R antagonist PD123319 (Fig 5C). Quantification of MCL-1 expression using ImageJ (~70 cells per treatment) showed that the NP-6A4-mediated increase in MCL-1 expression in HL-1 was significant (Fig 5D). Since NP-6A4-mediated increase in MCL-1expression is suppressed by the AT2R antagonist PD123319 (Fig 5D), increase in MCL-1 expression by NP-6A4 is mediated by AT2R. There was no significant difference between the MCL-1 expression levels of DMSO-treated and PBS-treated control cells.

### Effect of β-blockers on cell size and MCL-1 expression in hCAVSMCs

**Fig 6:**
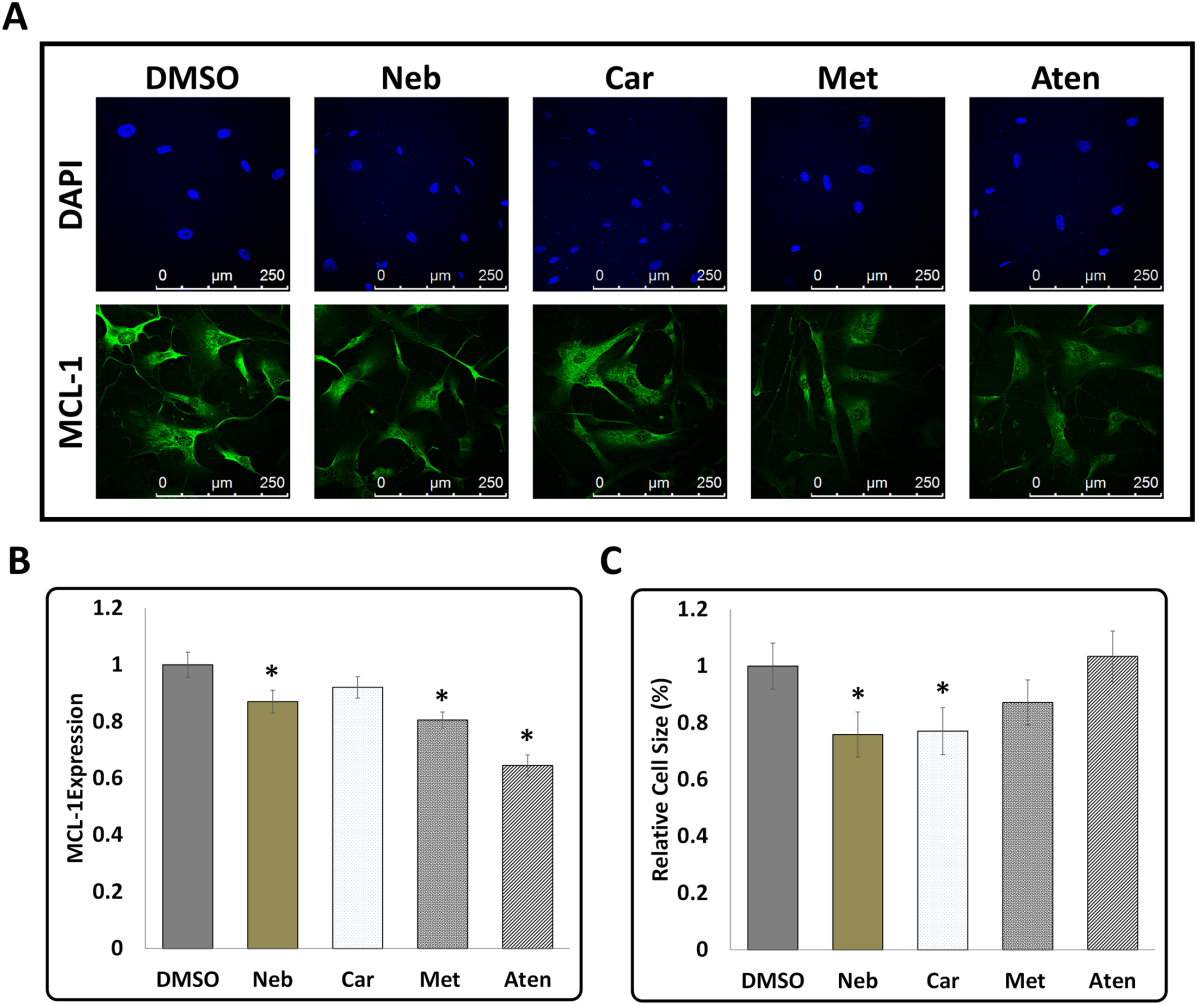
Effect of β-Blockers on MCL-1 expression and cell size in Human Coronary Artery Vascular Smooth Muscles Cells. **(A)** Representative images of immunofluorescence staining with anti-MCL-1 antibody and nuclear stain DAPI in hCAVSMCs in response to treatment by β-Blockers for a period of 6 hours (scale bars = 250 μm). **(B)** Quantification of MCL-1 expression in hCAVSMCs showed significant suppression of MCL-1 expression in response to treatment with Neb, Met and Aten **(C)** Quantification of cell size of hCAVSMCs by WGA staining showed significant suppression by Neb and Car but not Met and Aten. Data is shown as means ± SEM, n≥70 and *p<0.05 compared to control (DMSO) as determined by ANOVA followed by LSD post-test.

To determine whether the cell responses of mouse cardiomyocyte HL-1 cells to β-blockers are similar to that occur in other cardiovascular cells, we examined the changes in cell size and MCL-1 expression in primary cultures of nutrient-stressed hCAVSMCs in response to treatments with β-blockers. hCAVSMCs were nutrient-stressed via maintenance in Medium 231 without SMGS and then subjected to a 6-hour treatment with β-blockers as described for HL-1 cells. Immunofluorescence analysis using anti-MCL-1 antibody showed that MCL-1 expression was significantly suppressed in hCAVSMCs when exposed to Neb, Met and Aten (Fig 6A and 6B). Though treatment with Car also showed a trend towards suppression of MCL-1, this was not statistically significant. WGA staining showed that Neb was most effective in suppressing cell size in hCAVSMCs (~25%: n = 83 cells) (Fig 6C). Car also significantly reduced cell size (~23%: n = 74 cells). Treatment with Met also showed a trend towards reducing cell size, however this was not statistically significant (~13%: n = 108 cells). Aten treatment did not show any reduction in cell size. Thus, Neb treatment was most effective in reducing cell size in both mouse HL-1 cells and primary cultures of hCAVSMCs.

### NP-6A4 increased cell viability and expression of MCL-1 in primary cultures of hCAVSMCs

**Fig 7:**
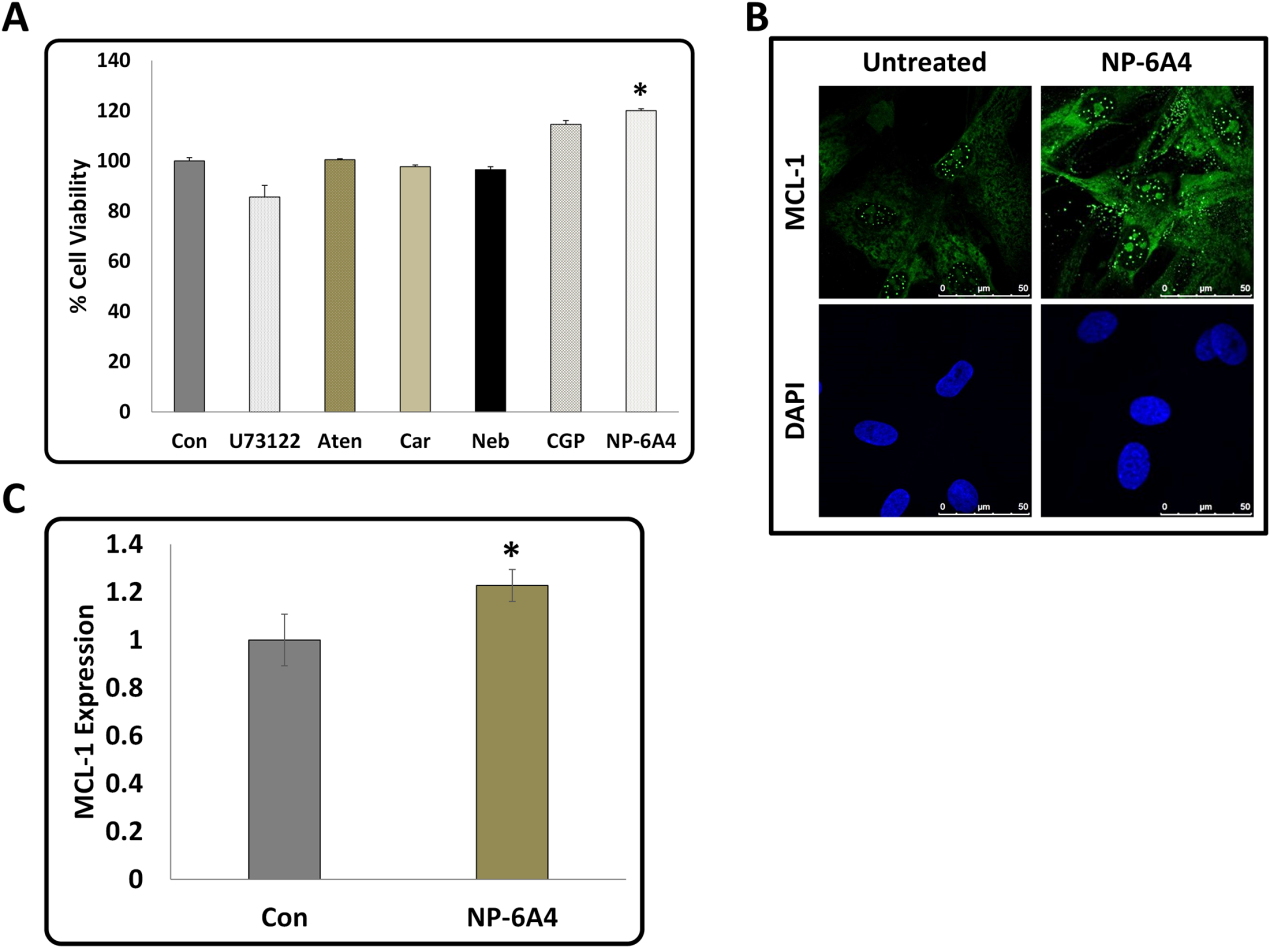
Effect of β-Blockers and AT2R agonists on cell viability and effect of novel AT2R agonist NP-6A4 on MCL-1 expression in Human Coronary Artery Vascular Smooth Muscles Cells. **(A)** Graph shows data from Cell the cell viability assessment using MTS proliferation assay kit. Treatment with β-Blockers did not significantly alter cell viability of hCAVSMCs while treatment with NP-6A4 resulted in the highest increase in the number of the viable cells. Data presented as means ± SEM, n≥3 and *p<0.05 compared to control **(B)** Representative images of immunofluorescence staining with anti-MCL-1 antibody and nuclear stain DAPI in hCAVSMCs in response to treatment by NP-6A4 (scale bars = 50 μm). **(C)** Quantification of MCL-1 expression in hCAVSMCs. Data is shown as means ± SEM, n≈20 and *p<0.05 compared to control as determined by Student’s 2-tailed T-test. Thus, NP-6A4 mediated increase in MCL-1 expression is a common signaling mechanism in mouse HL-1 cardiomyocytes and hCAVSMCs.

To determine whether NP-6A4 treatment could improve cell viability compared to β-blocker treatment in hCAVSMCs, we determined cell viability of nutrient-stressed hCAVSMCS in response to treatments with NP-6A4 and 2nd and 3rd generation β-blockers by MTS cell proliferation assay. Treatments with β-blockers did not significantly affect cell viability of hCAVSMCs (Fig 7A). Only NP-6A4 significantly improved cell viability of hCAVSMCs among the drugs tested (Fig 7A). Determination of MCL-1 expression in hCAVSMCs and quantification using ImageJ (~25 cells per treatment) showed that NP-6A4 increased MCL-1 expression in these cells by ≥22% (Fig 7B and 7C). Thus, NP-6A4-mediated up-regulation of MCL-1 expression is a signaling mechanism present in both mouse cardiomyocyte HL-1 cells (Fig 5C and 5D) and primary culture of human CAVSMCs (Fig 7B and 7C).

## Discussion

Nutrient starvation underlies cell death associated with ischemic heart disease, the leading cause of death worldwide [1–4]. Previous studies have shown that β-blockers are effective in mitigating cardiomyocyte apoptosis and they are the standard of care to reduce infarct size after cardiac ischemia. Aten and Met are 2^nd^ generation cardioselective β-blockers that reduce cardiomyocyte apoptosis caused by conditions such as coronary microembolization and exposure to lipopolysaccharides (LPS) [7, 8]. Car and Neb are 3^rd^ generation β-blockers with additional vasodilative effects [9, 10, 17]. However, differences in their impact on cell survival under nutrient starvation are not fully elucidated. We took advantage of the powerful xCELLigence RTCA system that can generate a CI signature that captures minute changes in the physical properties of cells such as size and adhesion as well as cell growth for each drug to investigate their effects [47]. Data presented here suggest that the CI signatures of Neb and Car are different from those of Aten and Met, and also different from each other. This is not surprising since Neb and Car are structurally different from the 2^nd^ generation drugs Aten and Met [9, 17]. Neb is also different from Car in that it is the only β-blocker that is both highly β1-selective, promotes endothelium-dependent vasodilation and also activates β3-AR [10, 17]. Thus the observation that they have unique CI signatures is in agreement with their differential structural and functional properties. Importantly, inhibition of any of the β-ARs resulted in the suppression of CI of HL-1 cells and this effect was exacerbated in response to double blockade of β-1 AR and β-3 AR. Therefore, CI serves as a useful physical measurement to determine the welfare of the cell in nutrient starvation. Previous studies have shown that reduction in CI correlates with poor cell survival [47]. Indeed treatment with phospholipase C inhibitor U73122, that is reported to increase apoptosis, was a powerful suppressor of CI in HL-1 cells in this study. The MTS cell proliferation assay did not show a significant suppression of cell viability (or growth) by any of the β-blockers and the extent of reduction in cell viability in response to U73122 assessed by MTS assay was lower compared to the extent of reduction of CI. Therefore, CI was more sensitive than the MTS assay in identifying the toxic effect of U73122 on HL-1 cardiomyocytes.

Consistent with the observation that ß-blockers reduce apoptosis of cardiomyocytes, we did not see any significant changes in the cell viability of mouse HL-1 cardiomyocytes or human CAVSMCs with different ß-blockers in the MTS cell proliferation assay. However, inhibition of phospholipase Cγ by U73122 significantly suppressed cell viability in hCAVSMCs. Thus, cell viability responses of mouse cardiomyocyte HL-1 cells and human CAVSMCs to β-blockers and U73122 were similar. Except Aten, other β-blockers reduced cell size of both HL-1 cells and hCAVSMCs. Since cell size is an important component of CI, we posit that reduction in cell size could have contributed to the reduction in CI induced by Neb, Met and Car.

Importantly, AT2R activation increased CI of serum-starved HL-1 cells. Ang II-mediated increase in CI was not suppressed by AT1R antagonist losartan, but was suppressed by AT2R antagonist PD123319. Both partial AT2R agonist CGP42112A and novel AT2R agonist NP-6A4 improved CI of serum starved HL-1 cells. Interestingly, NP-6A4 was more effective than CGP42112A in increasing the CI of serum-starved HL-1 cells (increase in CI:CGP42112A (≥14%), NP-6A4 (≥25%)) while pretreatment with PD123319 inhibited NP-6A4-induced increase in CI. MTS assay further showed that NP-6A4 rendered a significant increase in cell viability to both mouse HL-1 and human CAVSMC cells. Thus, NP-6A4, acting through AT2R, improved cell survival of both mouse cardiomyocyte HL-1 cells and human CAVSMCs more effectively than all other drugs tested here.

MCL-1 is critical for cardiomyocyte survival under stress and prevents vascular smooth muscle apoptosis [36–38]. Our results show that β-blockers suppress MCL-1 expression in HL-1 cells. MCL-1 may play a role in cell adhesion since it was reported that deletion of Mcl-1 could cause peri-implantation embryonic lethality and Mcl-1 −/− blastocysts failed to attach *in vitro* [40]. Therefore, we speculate that suppression of MCL-1 by β-blockers during the 6-hour treatment could have resulted in partial suppression of HL-1 cell adhesion and this effect could have contributed to the β-blocker-induced reduction in the CI. Interestingly, AT2R agonist NP-6A4 was very effective in increasing MCL-1expression in HL-1 cells as detected by immunofluorescence (Fig 5). Since MCL-1 promotes cell survival, regulation of MCL-1 expression by β-blockers and NP-6A4 may also be a contributing factor to the change in CI caused by these drugs. The observation that the AT2R agonist PD123319 inhibited the NP-6A4-induced increase in MCL-1 expression is consistent with the idea that NP-6A4 acts via AT2R to increase in MCL-1 expression. To date, there are no studies that show AT2R is a positive regulator of cardioprotective MCL-1. Comparison of the effects of β-blockers versus NP-6A4 of on cell viability and MCL-1 expression in human CAVSMCs and mouse cardiomyocyte HL-1 cells by β-blockers and AT2R agonists is summarized in Table 2.

**Table 2:** Comparison of the effects of cardioprotective drugs used in this study on cell index, cell viability and MCL-1 expression of cardiomyocytes.

Drugs and doses	Targets	CI (HL-1)	Viability (%)		MCL-1 (%)	
			HL-1	hCAVSMC	HL-1	hCAVSMC
		Values compared to untreated				
Nebivolol, 3μM	β1 AR; β3 AR	Lower, with dip	*96%	*96%	^#^71%	^#^87%
Carvedilol, 3μM	β1 and β2 AR	Lower, with dip	*93%	*98%	^#^75%	*92%
Metoprolol, 3μM	β1 and β2 AR	Lower	*93%	--	^#^68%	^#^80%
Atenolol, 3μM	β1 and β2 AR	Lower	*104%	*100%	^#^64%	^#^64%
Ang II 300nM	AT1R & AT2R	Higher	*110%	--	--	--
CGP42112A, 300nM	AT2R	Higher	*107%	^#^114%	*95%	--
NP-6A4, 300nM	AT2R	Highest	^#^120%	^#^120%	^#^125%	^#^123%

*Not statistically significant

^#^p<0.05

The AT2R partial agonist CGP42112A did not significantly increase MCL-1 in HL-1 cells. We have shown previously that interaction of CGP42112A with AT2R is different from that of Ang II [48, 49]. Mutation of Asp297 in the 3^rd^ extracellular loop abolished Ang II binding to the AT2R, but only partially reduced CGP42112A binding to the AT2R [48]. Similarly, truncation of C-terminus of the AT2R actually increased the affinity of CGP42112A whereas it partially suppressed the affinity of Ang II to the AT2R [49]. Since NP-6A4 increased MCL-1 expression and CGP42112A was not effective in this signaling, we propose that the mode of action of NP-6A4 is different from that of CGP42112A. Our observation that NP-6A4 could increase MCL-1 expression in human CAVSMC indicates that AT2R-mediated activation of MCL-1 expression is a mechanism that exists in both mouse and human cells. NP-6A4 is a patent-pending drug from Novopyxis Inc., and its pharmacological properties are not characterized. However, our results strongly suggest that NP-6A4 exerts its effects via AT2R and promotes cardiovascular cell survival under nutrient stress effectively.

One limitation of this study is that HL-1 cells do not represent all features of primary cultures of cardiomyocytes since HL-1 is an immortalized cell line. Additional studies are needed to determine whether NP-6A4 is capable of activating the AT2R-MCL-1 axis in primary cultures of human cardiomyocytes. However, our observation that NP-6A4 increases MCL-1 expression in hCAVSMCs implies that NP-6A4-mediated improved survival of cardiovascular cells during nutrient stress could be a common mechanism in mouse and human cells. In this study, our focus was on identifying the subtle differences between different cardioprotective drugs in improving cell survival. Apart from nutrient stress, hypoxia is another major component of ischemia-associated cell death [50, 51]. During hypoxia, mitochondria act as oxygen sensors and contribute to the cell redox potential, ion homeostasis, and energy production [51]. Since MCL-1 is known to localize to the mitochondrial matrix and couples mitochondrial fusion to respiration, it is conceivable that a drug that could promote MCL-1 expression such as NP-6A4 could be protective for cardiovascular cells in hypoxia stress. Additional studies are needed to determine whether NP-6A4 mediated up-regulation of MCL-1 would have cardiovascular protective effects in conditions of hypoxia.

## Conclusion

Loss of cell viability is a critical factor in cardiac damage resulting from ischemia. Stress caused by nutrient starvation is one of the critical components of ischemia-associated complications. Although there are several cardioprotective drugs that can reduce cell death, mechanisms by which they impact viability of cells dealing with nutrient starvation is not fully elucidated. Identifying the best drug that can promote cell viability during nutrient starvation will provide valuable insight into enabling reduction of cardiac damage. In this study we used cell index (CI) determination by the Xcelligence Real-Time Cell Analyzer (RTCA) as a method to uncover differences between widely used, and novel, cardioprotective drugs on mouse cardiomyocyte HL-1 cell survival subjected to a short term (6 hour) nutrient starvation. Data presented here show that 2^nd^ and 3^rd^ generation β-blockers have unique CI signatures and that all of them reduce rate of change in CI with time. It was interesting to note that Met, Car and Neb could reduce cell size of HL-1 cells and hCAVSMCs. Moreover, β-blockers also reduced expression of cardiovascular protective MCL-1 in HL-1 cardiomyocytes and hCAVSMCs. Thus, reduction in cell size and/or MCL-1 expression could have contributed to the reduction in CI of HL-1 cells caused by β-blockers. Conversely, Ang II increased the rate of change in CI with time and this effect was not mediated via the AT1R, but via the AT2R. Importantly, we show that a novel AT2R agonist NP-6A4 was most effective in increasing CI, cell viability, and expression of the anti-apoptotic protein MCL-1 in HL-1 cardiomyocytes. These data show for the first time that AT2R activation increases anti-apoptotic MCL-1 in cardiomyocytes. Interestingly, the partial agonist CGP42112A was not effective in increasing MCL-1 in cardiomyocytes. Collectively, these data suggest that NP-6A4-mediated AT2R activation is more protective for cardiomyocytes during nutrient starvation compared to other drugs tested in this study. Finally, these results also confirm that AT2R-meidated increase in MCL-1 expression by NP-6A4 (the AT2R-MCL-1 axis) also occurs in human CAVSMCs. Thus, the AT2R-MCL-1 axis is a common protective mechanism that improves cell viability in mouse and human cardiovascular cells.
